# Scientific Items

**Published:** 1888-09

**Authors:** 


### SCIENTIFIC ITEMS.

**The Home of the Great Auk**.—That strange bird, the auk, who had an immense body with wings so puny they would not suffice to lift it from the ground, is now extinct, or rather it is believed to be, and all that remains to remind us of its one-time existence are its bones and some aged and ill-looking stufled specimens or reproductions in the various museums; the same being in no two cases alike, neither as to shape, arrangement of plumage, nor general expression. and so it is we get only a confused idea of how the bird really looked. Funk Island, an exposed rock, thity-two miles northeast of Cape Freels, Nova Scotia, was once famous as the resort of the great auk. Here these curious birds gathered in multitudes, and in the breeding season, the weather being clear—a rare condition be it said—were often seen from the open sea, standing in serried ranks, like line after line of an army drawn up in battle array; tall, gaunt, silent.

Last summer, Mr. Frederick Lucas visited this island, a boat being sent with him and some companions from the Fish Commission's schooner Grampus; the weather being selected with care and arrangements made to camp, for the island can only be approached or quitted under most favorable conditions, so exposed it is, with a heavy surf on its seaward face and threatening rocks and angry currents to leeward. The whole island was found to be strewn with the bones of these birds; they had been slaughtered by the hundreds, perhaps by thousands, for they could neither fly nor fight, but only waddle slowly along wheresoever they might be driven. An inclosure was found, built of stones—a sort of shambles into which they were evidently driven to be despoiled of their feathers, though it is certain they were sometimes driven aboard boats over planks laid from gunwale to shore. Over a hundred sets of bones were gathered, the crania of each specimen showing signs of fracture, as if they had been beaten about the head with clubs.—*Ib.*

**"Self-Mending Snakes."**—Dr. John J. Taylor, Streator, Ill., says: In the April number of the *Illustrated Medical Journal* an article appeared under the above caption—signed Oliver White, Secretary of the Peoria Scientific Association. On reading the article the first thought was: "All Fools' Day." The statement that the glass or jointed snake, a species of the lizzard family, has the power to replace separated segments of his vertebral column, has appeared several times in the metropolitan papers, and a goodly number of writers have asserted the same thing as O. W. I have been a resident of Illinois for thirty years, and when I first came to this county (La Salle) the prairies were fairly alive with snakes, and the glass snake possessed a fair share of the domain. The stories were numerous of the powers of the joint snake, but few doubting the tale. We boys had quite a curiosity to know whether there was any truth in the thing, and repeatedly tried the little jokers, but invariably found that his snakeship would never stick his broken segments back again. In almost every instance the ants appeared by the second day, and kept at work until nothing but the whitened bone remained to tell the sad tale. Further, I have often seen the glass snakes when so injured, with their stub tails healed, and have handled "lots" of them.

**Ice on Mars**.—At a recent meeting of the Academy of Sciences, Paris, M Janssen, president, in the chair, observations were made on the canals of the planet Mars, by M. Fizeau. The various circumstances connected with these appearances, as lately described by MM. Perrotin and Schiaparelli, suggest a strong analogy with ce tain phenomena of glaciation—parallel ridges, crevasses, rectilinear fissures, often of great length and at various angles—observed in the regions of large glaciers in Switzerland and especially in Greenland. This leads to the hypothesis of a vast development of glaciation on the surface of Mars, where, the seasons being relatively longer and the temperature much lower, the conditions must also be more favorable than on the earth for these manifestations. The reading of the paper was followed by some remarks by M. J. Janssen, who gave a guarded assent to M. Fizeau's "very ingenious and very beautiful" theory.—*Scientific American.*

**The Mosquito a Blessing to Man.**—A lecture was recently delivered at Madras, India, on that interesting and familiar pest, the mosquito. The lecturer, Mr. H. Sullivan Thomas, asserts that it is only the female mosquito that does the biting. He considers the mosquito a most useful pest, seven-eighths of its existence being devoted to the service of men and only one-eighth to their annoyance. It exists in the larval state twenty-one days, and during that period engages in sanitary work with ardor and thoroughness. Wherever there is dirty water, wherever there is a filthy drain, there the mosquito larvæ are to be found in hundreds. voraciously devouring the contaminating matter.—*New Orleans Times-Democrat.*

**Twins, one White and one Black.**—Dr. Newton Hill, of Pickensville, Ala., sends to the *Medical and Surgical Reporter* the following report of a case: "A young negro girl, about eighteen years of age, gave birth to twins at seven months, one of which was as black as the *ace of spades*, and the other as white as any white child I ever saw. This child has been engaged as nurse in a white family a part of a year, but she has associated with white and black. Both cords were attached to the same placenta. Is this merely a freak of nature, or is it possible that they have different fathers? I would like to have the opinion of some of the brethren."—*Illustrated Medical Journal.*

**A Sympathetic Heart.**—Old Mrs. Bently: "I felt so sorry for a poor man to-day, Josiah. He told me he had been deaf and dumb all his life, and I gave him a dime." Old Mr. Bently: "How could he be dumb an' tell ye that he was dumb?" Old Mrs. Bently: "Why, deary me, Josiah Bently, I never thought o' that."—*The Epoch.*

